# Serious shortcomings in assessment and treatment of asylum seekers’ mental health needs

**DOI:** 10.1371/journal.pone.0239211

**Published:** 2020-10-07

**Authors:** Amand Führer, Andreas Niedermaier, Vivian Kalfa, Rafael Mikolajczyk, Andreas Wienke

**Affiliations:** 1 Institute of Medical Epidemiology, Biometrics and Informatics, Martin-Luther-University Halle-Wittenberg, Halle (Saale), Germany; 2 Psychosocial Centre for Refugees and Victims of Torture (PSZ), Halle (Saale), Germany; Medical University of Vienna, AUSTRIA

## Abstract

**Background:**

The prevalence of psychological complaints is known to be very high in populations of asylum seekers. Despite this, data on the health care system’s ability to adequately meet these high-risk populations’ mental health needs are scarce. This article investigates how well the German outpatient health care system is able to detect and adequately treat them.

**Methods:**

To this end, we combined data from a cross-sectional survey with billing data from the local social welfare office from the year 2015. Using descriptive statistics, the data of the cross-sectional study are used to quantify the psychological health care *needs* of asylum seekers while the secondary data analysis indicates the actual access to and extent of psychological *treatment*.

**Results:**

In the cross-sectional study, 54% of patients were screened positive for symptoms of depression, 41% for symptoms of anxiety disorder and 18% for symptoms of Posttraumatic Stress Disorder. In total, 59% were screened positive for at least one of these three disorders. However, when contrasting these screening-based prevalences with the prevalences based on data from the health care system, a mismatch becomes apparent: According to the social welfare office’s billing data, only 2.6% of asylum seekers received the diagnosis of depression, 1.4% were diagnosed with anxiety disorder and 2.9% with Posttraumatic Stress Disorder (PTSD). In combination, 4.9% were diagnosed with at least one of these three disorders. Overall, less than one tenth of asylum seekers with symptoms of depression, anxiety or PTSD received the corresponding diagnosis by the health care system. Among those who were diagnosed, about 45% received no treatment at all, while 38% were treated with drugs alone. Only 1% of all patients received psychotherapy.

**Conclusions:**

Psychological complaints are very common among asylum seekers, yet only a small proportion of this population receives the corresponding diagnoses and treatment. While various factors can contribute to these shortcomings, there is an urgent need to systematically address this deficit and introduce measures to improve mental health care for this high-risk population.

## Introduction

A vast number of studies show that psychological problems are highly prevalent in refugee populations all over the world [1–3] and also in Germany [4,5] (for an explanation of our terminology see Supplement 1). The reasons for this are manifold and well established: While many asylum seekers are victim to potentially traumatizing experiences in their countries of origin and suffer from adversity that made them leave their home in the first place [6,7], many experience further violence, abuse and insecurity during their flight [8,9]. Migration studies speak of pre- and peri-migratory stressors here [10,11].

However even after asylum seekers’ arrival in Europe, their psychosocial situation remains burdensome. These post-migratory stressors include uncertainty concerning the outcome of asylum claims [12–14], poor living conditions in shelters with no privacy and often substandard housing [14–16], social marginalization and legally restricted possibilities for family reunion [11], economic hardships due to legal restrictions of access to the labor market and social benefits [17], and the experience of racism and violence [15,18,19].

Considering these facts, facilitating asylum seekers’ access to adequate psychological care seems a common sense measure. Accordingly, attempts to facilitate access to care have been incorporated into international law: In 2011 the European Parliament urged its member states to “focus on the needs of vulnerable groups, including disadvantaged migrant groups” [20] while the EU’s Directive 2013/33/EU explicitly specifies for migrants with chronic mental conditions that “Member States shall provide necessary medical or other assistance to applicants who have special reception needs, including appropriate mental health care where needed” [21]. The EU Directive 2013/33/EU was signed by the German government in 2013 and became national law in July 2015.

Subsequently, institutions such as the German National Academy of Sciences [22], the German Association of Psychosocial Centres for Refugees and Victims of Torture [23], and national guidelines [24] outlined in great detail means to achieve these aims. Still, scientific studies and reports of civil society alike regularly illustrate that asylum seekers’ access to psychological care continues to be alarmingly underdeveloped. Many patients are only able to find treatment outside the regular health care sector, facilitated by NGOs and other members of civil society [25].

The extent of the presumed health care gap for psychological treatment is so far unknown. Therefore, the aim of this paper is to investigate the extent of this presumed gap. In doing so, we found substantial deficits both in diagnostics and guideline-adherent therapy of psychological complaints.

## Materials and methods

### Study population

After their arrival in Germany, asylum seekers are assigned to a federal state and its reception center, where they go through the first stages of their asylum procedure. Depending on their country of origin, they stay at the reception center until the asylum process is completed (this is usually the case for people from a country with a low probability of asylum approval), or they are transferred to a municipal shelter where they complete the remainder of the process [26]. During this time, they have a particular legal status: They are not allowed to work, their access to social benefits is very limited and often restricted to vouchers and/or benefits in kind, and they have no access to the statutory health insurance [13,27]. Instead, their medical needs ought to be covered by §§ 4 and 6 of the Asylum Seekers’ Benefits Act (ASBA), which grants a high degree of discretionary power to the local social welfare office (*Sozialamt*) [28], the government agency responsible for the payment of welfare subsidies and for the provision for asylum seekers. As a result, in most municipalities the extent of medical services covered by the social welfare office is considerably smaller than for regularly insured patients [27]. After 15 months in Germany, asylum seekers usually become entitled to statutory health insurance, irrespective of their legal status. As of now, this period has been extended to 18 months.

The asylum seekers included in this study have already been transferred to the municipality’s responsibility and mostly live in shelters. All of them are receiving benefits according to the ASBA and are still waiting for the approval of their asylum claim.

### Study design

This analysis is based on two sources of data: a secondary data analysis of the local social welfare office’s billing data concerning all asylum seekers registered in 2015 in the city of Halle (Saale), Germany, and a cross-sectional study involving a subpopulation of those asylum seekers. The data of the cross-sectional study are used to quantify the psychological health care *needs* of asylum seekers, while the secondary data analysis indicates access to and extent of psychological *treatment*.

We restricted the analysis of treatment to the outpatient sector, since there is a consensus that patients with the conditions in question are to be treated as outpatients and that inpatient treatment in these cases signals shortcomings in the outpatient sector [29].

### Cross-sectional survey

In August 2015, a sample of 214 respondents was randomly recruited out of 560 eligible asylum seekers in Halle (Saale), Germany. Participants were approached in their shelters by going from door to door. A week before the survey all eligible asylum seekers (or their parents or guardians in the case of minors) had already been informed about the study by a social worker who handed them a document explaining the study aims and matters of data protection and voluntariness in the asylum seekers’ respective mother tongue. On the day of the survey, these issues were once again explained in the respondents’ mother tongue by native-language study assistants. Then, they were asked to verbally give informed consent on their participation and fill in the questionnaire. (For participants under the age of 18 years (the age of consent in Germany), a parent or other guardian had to agree to the study participation as well.) Except for two people, who refused to participate due to time constraints, all approached eligible asylum seekers agreed to take part in the survey. To be included in the study, participants had to be at least 16 years of age and speak Arabic, Farsi, French, Hindi, or English. Participants anonymously filled in a questionnaire in their mother tongue that contained two psychometric screening tools measuring symptoms of depression and anxiety (Hopkins-Symptom-Checklist 25, HSCL-25) [30], and symptoms of PTSD (fourth part of Harvard Trauma Questionnaire, HTQ) [31]. Both instruments have been shown to perform well in cross-cultural settings in earlier studies [32–34]. The HSCL-25 was analyzed separately for symptoms of depression and symptoms anxiety disorder, using a cut-off value of >1.75 for each score [35]. For the HTQ, values >2.5 were considered indicative of PTSD [36].

More information on methodic details can be found in [5].

### Billing data

Billing data was gathered from the local social welfare office and digitalized. Hereby information on the billing physicians and their specialty, as well as diagnoses (as ICD-code), treatment procedures (as EBM-codes), prescribed medicines (as ATC-codes) and the costs for each were documented. The billing data covered the entire population of registered asylum seekers in Halle (Saale) during the year 2015 and also *included* those individuals sampled in the cross-sectional survey. To ensure comparability of the data sets, only asylum seekers of at least 16 years of age were included in the analysis of the billing data.

Since the screening tools employed in the cross-sectional study measure symptoms that might also occur in disorders other than depression, anxiety disorder and PTSD, we grouped the ICD-codes that could have been assigned by physicians according to the *phenomenology* of the diseases by taking into account if the respective diagnosis makes it likely that a patient would at some point in his or her history be screened positive with the tools we employed: In the following, the physicians’ diagnosis of “depression” subsumes the ICD-codes F25.1, F31.3–6, F32, F33, F34, F38, F39, F92.0, “anxiety disorder” includes F40 and F41, and “PTSD” corresponds to F43.

The billing physician’s specialty was documented using the physician’s ID assigned by the Association of Statutory Health Insurance Physicians which includes information on a physician’s specialty. Specialties were then grouped into broader categories: Different types of family doctors were included in one group and all specialties concerned with psychological complaints were categorized as “psychiatric specialist”. The latter group includes the specialties “neurology and psychiatry”, “psychiatry and psychotherapy”, “forensic psychiatry”, “psychosomatic medicine and psychotherapy”, “medical psychotherapy”, “psychological psychotherapy” and “pediatric psychotherapy”.

In classifying the therapies offered to patients with diagnosed depression, anxiety disorder or PTSD, we distinguished between psychotherapy and verbal intervention (*Gesprächsintervention*), where verbal intervention refers to a consultation of at least 15 minutes with a physician who underwent training for mental basic care. While psychotherapy is offered only by the above-mentioned psychiatric specialists, verbal interventions can be conducted by any medical specialty.

Prescribed medicines were categorized according to their ATC-code. The following groups of codes on the four-digit level of the ATC-classification are potentially indicated for treating symptoms of depression, anxiety disorder or PTSD: N05AN, N05B, N05C, N06A, N06C. Medicines belonging to these groups are hereinafter referred to as “specific drugs”.

### Estimating the extent of the health care gap

We use the findings from the cross-sectional study to calculate the proportion of asylum seekers with psychological symptom-scores above the cut-off value. Since studies have shown that among the regularly insured population only about 50% of positively screened patients are later diagnosed with depression by their family doctor [37], it is likely that not all patients screened positive would need therapy. Nevertheless, all of them show distinct symptoms that warrant further investigation. Therefore, we interpret this proportion as the percentage of asylum seekers that would need to be examined by a physician *at least once* in order to confirm or refute the results found in the screening.

We then compared this proportion derived from the cross-sectional study with the combined prevalence of diagnoses, refuted diagnoses, and diagnostic and therapeutic procedures related to depression, anxiety disorder or PTSD as gathered from the social welfare office’s billing data. From this comparison we estimate how well the health care system is able to *detect* asylum seekers with psychological complaints.

In a second step, we investigated the kind of treatment that patients who were diagnosed with one of the three conditions received within the outpatient health care system. This serves to assess how well the health care system is able to *treat* asylum seekers with psychological disorders.

### Statistical analysis

Descriptive statistics are reported as means and absolute and relative frequencies with their corresponding 95% confidence intervals.

### Ethics

The cross-sectional part of the article underwent ethics clearance and was approved by the institutional review board of the Faculty of Medicine at Martin-Luther-University Halle-Wittenberg, Germany (Nr.: 2015–74).

The secondary data analysis performed as part of this study uses administrative data which fulfils all necessary requirements of the Federal data protection act of the Federal Republic of Germany. As the data is fully anonymous and did not involve any experiments, according to national guidelines (AGENS (2014): Gute Praxis Sekundärdatenanalyse (GPS): Leitlinien und Empfehlungen) no ethics clearance for this part of the investigation was necessary.

## Results

A total of 4 107 people were registered as asylum seekers at the social welfare office in Halle (Saale), Germany in the year 2015, of whom 3 388 (83%) were above the age of 15 years. Those adult asylum seekers effected 12 944 billing documents in 2015. Median time of observation within the year 2015 was 113 days (min = 1, max = 365), which reflects that many asylum seekers went in and out of entitlement during the one-year observation period.

The comparison of the population sampled for the cross-sectional survey to the entire cohort of asylum seekers shows a slight over-representation of male and Syrian asylum seekers in the cross-sectional survey (due to language being a selection-criterion for the survey) as well as a higher proportion of married respondents. More details of the demographic properties of the study populations are shown in Table 1.

**Table 1:** Demographic characteristics of the cross-sectional sample and the whole cohort of asylum seekers in 2015 in Halle (Saale), Germany.

		cross-sectional sample N = 214		whole population > 15yrs N = 4 107	
		n	%	n	%
Age [years]	16–24	60	28	1228	36.3
	25–34	100	46.7	1314	38.8
	35–44	27	12.6	546	16.1
	≥45	14	6.5	300	8.9
	missing	13	6.1	.	.
	average:	29.16	range: 16–65	29.9	range: 16–89
Gender	male	182	85	2625	77.5
	female	24	11.2	763	22.5
	missing	8	3.7	.	.
Marital status	married	92	43.0	825	24.4
	single	115	53.7	2366	69.8
	divorced	2	0.9	24	0.7
	widowed	2	0.9	9	0.3
	unknown	3	1.4	162	4.8
Country of origin	Syria	145	67.8	1581	46.7
	Afghanistan	28	13.1	269	7.9
	Benin	11	5.1	157	4.6
	Iran	10	4.7	161	4.8
	India	5	2.3	103	3.0
	Other	15	7.0	1116	32.9

### Detection deficit

In the cross-sectional study, 54% of patients (n = 116; 95%-CI: 47.5%–60.9%) were screened positive for symptoms of depression, 41% (n = 87; 95%-CI: 34.1%–47.2%) for symptoms of anxiety disorder and 18% (n = 38; 95%-CI: 12.6%–22.9%) for symptoms of PTSD. Co-morbidity was high, so that in total 59% of all respondents (n = 127; 95%-CI: 52.8%–65.9%) showed symptoms of at least one of the three disorders.

When contrasting these estimates with the percentage of people diagnosed with psychological complaints within the health care system, a gap becomes apparent: Within the year 2015, only 2.6% (n = 105; 95%-CI: 2.1%–3.1%) of asylum seekers received a diagnosis of depression, of which most diagnoses (85%) were major depression (ICD-code F32), 9% were recurrent major depression (ICD-code F32) and 3% were schizoaffective disorders of the depressive type (F25.1).

**Fig 1:**
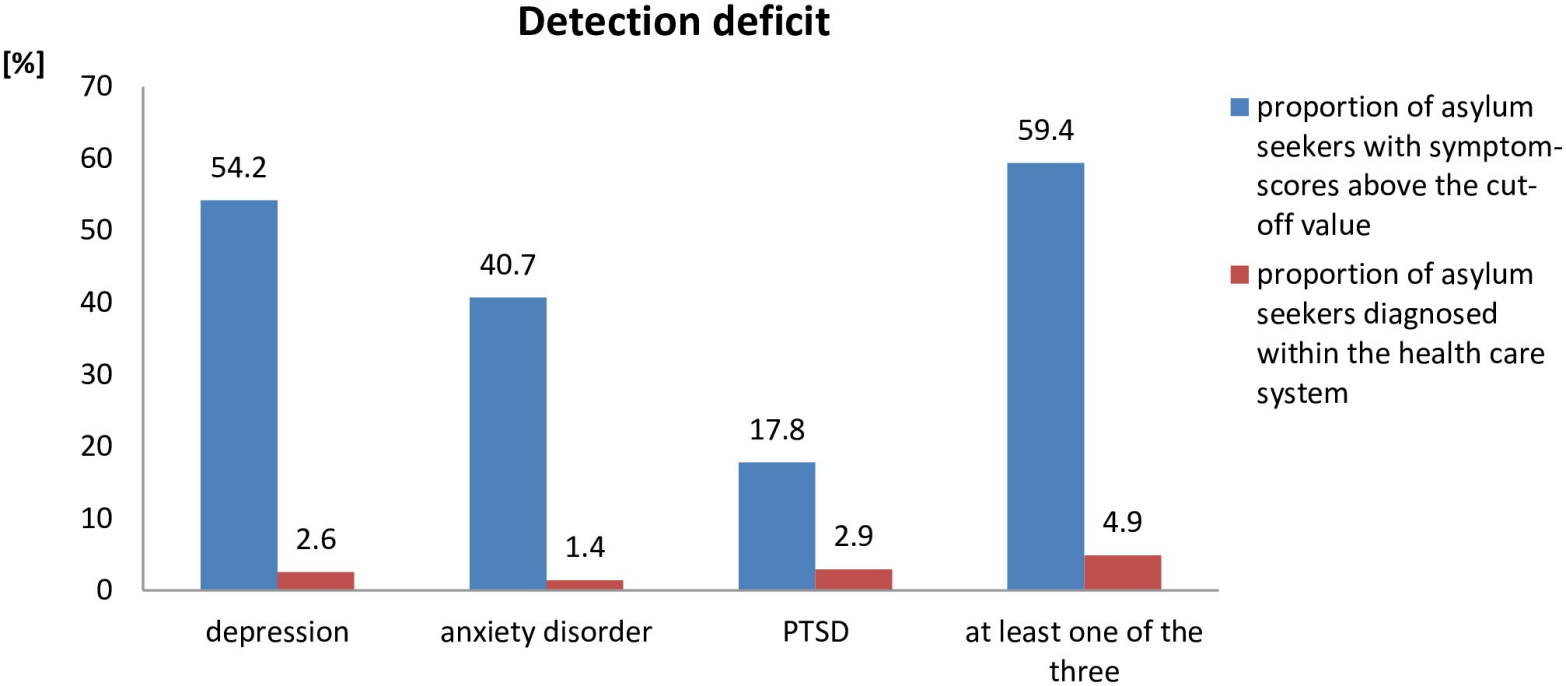
Comparison of the proportions of asylum seekers with psychological complaints in the cross-sectional study and in the outpatient health care system. The difference between the bars signals a detection deficit.

1.4% (n = 58; 95%-CI: 1.1%–1.8%) of asylum seekers were diagnosed with anxiety disorder and 2.9% (n = 121; 95%-CI: 2.5%–3.5%) with PTSD. 4.9% (n = 201; 95%-CI: 4.3%–5.6%) were diagnosed with having at least one of the three disorders. Fig 1 illustrates the magnitude of the detection deficit.

### Psychological care gap

#### A) Physicians’ specialties

Of the 201 patients diagnosed with at least one of the three disorders, 39% (n = 79) were treated for the disease by their family doctor alone, while 43% (n = 87) received care from a psychiatric specialist (alone or in combination with the family doctor and/or other specialties), and 11.4% (n = 23) from specialties other than family medicine and psychiatry. The remaining 6% (n = 12) were treated either in a hospital’s emergency department, or by a family doctor and another specialty in parallel.

#### B) Drug therapy

85.5% (n = 171) of all patients with at least one psychological diagnosis received medication, while 48% (n = 96) received drugs specific for the respective psychological complaints. Among these, antidepressants were the most common group (83% of all prescribed *specific* drugs), followed by anxiolytic substances (10%) and hypnotics and sedatives (7%). 80% of the specific drugs were prescribed by a psychiatric specialist, 18% by a family doctor and 2% by other specialties.

#### C) Psychotherapy

Only two patients received psychotherapy (1% of all patients with a psychiatric diagnosis). A larger number of patients (n = 33, 16.4%) was treated with verbal intervention. These 33 patients received a total of 56 sessions of verbal intervention. 71% of the verbal interventions were performed by family doctors, 5% by psychiatric specialists and 23% by other specialties.

#### D) Treatment options summary

Drug therapy and psychotherapy are considered the two main pillars of psychological treatment. In our study population, 38% (n = 76) of patients were treated using drug therapy alone, while 6.5% (n = 13) received a verbal intervention. 9.5% (n = 19) were treated with a combination of drug therapy and verbal intervention. 45% (n = 91) received no therapy for their psychological complaints at all.

When stratifying the treatment options according to diagnosis, the percentage of untreated patients varied: Among asylum seekers diagnosed with depression, 28% received no treatment, while 38% and 47% respectively of patients with anxiety disorder and PTSD went without treatment. More details are shown in Table 2.

**Table 2:** Treatment combinations stratified according to disease.

	Depression		Anxiety		PTSD		At least one diagnosis	
	n	%	n	%	n	%	n	%
Drug therapy only	53	50	23	41.8	47	38.8	76	37.8
Verbal intervention only	8	7.7	5	9.1	3	2.5	13	6.5
Psychotherapy only	0	0	0	0	1	0.8	1	0.5
Drug Therapy + Psychotherapy	0	0	0	0	0	0	0	0
Drug therapy + verbal intervention	14	13.5	5	9.1	12	9.9	19	9.3
Drug therapy + verbal intervention + psychotherapy	1	1.0	1	1.8	1	0.8	1	0.5
No specific treatment at all	29	27.9	21	38.2	57	47.1	91	45.3

## Discussion

This article presents two main findings: First, based on screening tests, a much larger proportion of asylum seekers experiences symptoms of various mental health disorders than are formally diagnosed. Second, among those diagnosed within the health care system, many receive no treatment at all and only very few patients receive psychotherapy. Thus, we conclude a substantial deficit in addressing this population’s mental health needs.

As outlined in the introduction, the high prevalence of psychological conditions among asylum seekers is well established in the literature [2–5,38] and estimated to be about five times the prevalence of the general population [39,40]. This article adds to the current state of knowledge by showing that the German health care system so far fails to adequately address this issue.

At first glance, one could argue that outpatient treatment for psychological complaints is generally problematic in Germany [41]. However, when comparing our findings to findings evaluating the situation for regularly insured patients with psychological complaints, it becomes apparent that the situation of asylum seekers is *exceptionally* dire: Whereas in regularly insured patients between 40% and 75% of e.g. patients with symptoms of depression receive the corresponding diagnosis in the health care system [40,42,43], in our study population this proportion was only around 5%. This might be explained by language barriers [4,10,44], socioculturally shaped presentations of symptoms unfamiliar to many physicians [2,4,45], or problems related to patients’ irregular health insurance status [46]. Also, asylum seekers’ problems with navigating through the health care system have been described as responsible for the observed low proportion of patients receiving mental health diagnoses [47].

Considering the high prevalence of psychological complaints in asylum seekers, the introduction of screening measures seems advisable. Indeed, the national guideline for depression recommends screening of high-risk populations [48], and the EU Directive 2013/33/EU explicitly demands the implementation of screening measures to identify “applicants with special reception needs” [21]. Our data strongly supports these demands and highlights the need for systematic implementation of procedures to early identify asylum seekers with psychological complaints and facilitate their referral to adequate care.

Yet not only the deficit in the identification of symptomatic asylum seekers is problematic; the treatment they receive once diagnosed seems to be insufficient as well. While virtually all regularly insured patients in Germany who suffer e.g. with depression receive either drug therapy, verbal intervention or both [40,49], in our cohort 28% of all patients with this diagnosis received no therapy at all. Furthermore, the comparably high proportion of depressed patients treated by drug therapy alone seems to be indicative of a deviation from medical guidelines, which discourage the use of antidepressant drugs as a first-line treatment for mild depression [48].

A somewhat surprising finding is the high proportion of patients treated by psychiatric specialists in contrast with the very low number of psychotherapies: Apparently, many psychiatric specialists abstain from offering guideline-adherent therapy and favor drug therapy over psychotherapy. While this might reflect a general tendency in the German health care system, where psychiatrists often focus on drug therapy and psychotherapies are delegated to other psychiatric specialists, other factors might be relevant as well. We propose that other reasons might be seen in difficulties with identifying the correct indication when symptom presentation differs from what is common in the physicians’ culture [44,50]. Another factor might be that interpreters are not readily available within the German health care system, and even if they are available cost absorption for interpreters is difficult and time-consuming [10,51–53]. Additionally, many psychiatric specialists might not be familiar with the treatment of complaints more common among asylum seekers, e.g. Posttraumatic Stress Disorder [8,54]. Also, the risk that the social welfare office might refuse to reimburse their costs might influence specialists and family doctors alike in the choice of treatment they offer to asylum seekers: It has been reported that up to 35% of applications for cost reimbursement for psychotherapy for asylum seekers are rejected [55], compared to only 1–3% in regularly insured patients [12].

In summary, asylum seekers’ mental health seems to be caught in a vicious trap: First, many asylum seekers are exposed to multiple stressors and violations before, during and after their flight. Second, irrespective of their high risk for mental illness, medical guidelines and legal obligations, asylum seekers are rarely screened for mental problems and as a result often do not receive the health care system’s due attention. Third, even if they are diagnosed with mental health problems, they often receive no treatment at all and if they receive treatment it is often substandard compared to regularly insured patients.

All three dimensions are man-made: Dangerous flight routes to Germany are largely a result of the EU’s border policy [56,57] and many of the post-migratory stressors directly result from political decisions on the federal, state or district level [58], while the administrative barriers in asylum seekers’ mental health care were explicitly intended when the ASBA was passed in 1993 [58,59].

Therefore, we think it justified to think of asylum seekers’ exposure to substandard mental health care as a form of structural violence. First introduced by sociologist Johan Galtung [60], this term describes how “historically given (and often economically driven) processes and forces […] conspire” [61] to cluster risk factors, morbidity and early death in some populations and not in others [62]. It highlights how social, political, economic and infrastructural factors can lead to bodily and psychological harm for particular groups of people, and further emphasizes that despite the efforts of individual agents to provide high-standard care, structural forces of exclusion are often difficult to overcome and thus should be the subject of concerted effort to change. Therefore, the pattern of undersupply that we show in our paper is more than a mere reflection of individual suffering—it highlights that there is a human rights issue at hand and that our society so far fails to live up to its promise of the “highest attainable standard of health” for all [63].

### Limitations

There are a number of limitations that necessitate a cautious interpretation of our findings: First of all, our estimation of the proportions in the cross-sectional sample is based on screening tools, which are reported to have a specificity of between 73% and 93% [34,64] and might therefore overestimate the prevalence of asylum seekers with mental illness. Also, studies have shown that—among regularly insured patients—for instance, only about 50% of patients screened positively for depression are later found to be depressed by family doctors [37]. Various reasons for this finding are discussed in the literature, among them the possible underdiagnosis of psychological disorders by family doctors due to their recourse to information not included in diagnostic manuals [43,65]. When interpreting our findings, it is therefore important to keep the difference between screening-based findings and diagnoses derived by a physician or a psychologist in mind.

In addition, the cross-sectional sample was recruited based on language and included only asylum seekers who were living in municipal shelters at the time of the survey. This might introduce a bias for the comparison with the billing data, which covers the unselected population of all asylum seekers.

Secondly, both datasets (from the cross-sectional survey and the billing data) were anonymous. Therefore we could compare only the aggregated data and were not able to link both datasets on the level of the individual patient. We are therefore unable to quantify how many of the individuals screened positive in the survey were detected by the health care system and which care they received.

Thirdly, the prevalence for depression estimated from the billing data might overestimate the true prevalence, since we counted all disorders as depression that might at some point give rise to symptoms of depression. Still, since the majority of diagnoses are major depressions we consider the potential for serious overestimation to be small.

Lastly, our analysis focused only on treatment within the health care system. The reason for this decision was twofold: First, since NGOs and other actors outside the health care system cannot bill the social welfare office for the treatments they offer, they do not appear in the secondary data available to us. Second, providing adequate medical care is the responsibility of the health care system and not of civil society. For this reason, excluding NGOs from our analysis was necessary in order to estimate the extent of the health care system’s failure to provide psychological care for all who need it.

## Conclusion

Psychological complaints are very common among asylum seekers. Still, only a small proportion of this population receives the corresponding diagnoses and treatment. While various factors can contribute to this gap, there is an urgent need to further assess this deficit and introduce measures to address it.

## Supporting information

S1 File (DOCX)
