# Supplementary material for: Serious shortcomings in assessment and treatment of asylum seekers’ mental health needs
Source: PLoS One. 2020 Oct 7;15(10):e0239211. doi: 10.1371/journal.pone.0239211 (PMC7540848; doi:10.1371/journal.pone.0239211)
Supplement: S1 File — (DOCX) [file pone.0239211.s001.docx]

The term *refugee* is often used in two ways: As a description of people who have biographical experience of flight irrespective of their legal status, and as a legal term for people whose asylum claims have been granted according to the Geneva Convention. We use the term in the first, more general, sense.

The term *asylum seeker* is a legal term that refers to people who claimed asylum but whose asylum procedure is still ongoing [6].
